# A template for the authoring of statistical analysis plans

**DOI:** 10.1016/j.conctc.2023.101100

**Published:** 2023-06-09

**Authors:** Gary Stevens, Shawn Dolley, Robin Mogg, Jason T. Connor

**Affiliations:** a DynaStat Consulting, Inc., 119 Fairway Court, Bastrop, TX, 78602, USA; b Open Global Health, 710 12th St. South, Suite 2523, Arlington, VA, 22202, USA; c Takeda Pharmaceuticals USA Inc., 95 Hayden Avenue, Lexington, MA, 02421, USA; d ConfluenceStat, 3102 NW 82nd Way, Cooper City, Florida, 33024, USA; e University of Central Florida College of Medicine, 6850 Lake Nona Blvd, Orlando, FL, 32827, USA

**Keywords:** Statistical analysis plan, SAP, Trial protocol, Prospective analysis

## Abstract

A number of principal investigators may have limited access to biostatisticians, a lack of biostatistical training, or no requirement to complete a timely statistical analysis plan (SAP). SAPs completed early will identify design or implementation weak points, improve protocols, remove the temptation for p-hacking, and enable proper peer review by stakeholders considering funding the trial. An SAP completed at the same time as the study protocol might be the only comprehensive method for at once optimizing sample size, identifying bias, and applying rigor to study design. This ordered corpus of SAP sections with detailed definitions and a variety of examples represents an omnibus of best practice methods offered by biostatistical practitioners inside and outside of industry. The article presents a protocol template for clinical research design, enabling statisticians, from beginners to advanced.

## Highlights

• Comprehensive template for biostatisticians: best practices, annotations, code samples, and more. Perfect for both early and mature statisticians.
• Promotes equity by bridging the gap for biostatisticians in low-resource settings, ensuring access to tools available in the Global North.
• Benefits of earlier SAP completion alongside the protocol are described.

## 1 Introduction

### 1.1 The statistical analysis plan

The Statistical Analysis Plan (SAP) is a key document that complements the study protocol in randomized controlled trials (RCT). SAPs are a vital component of transparent, objective, rigorous, reproducible research. The SAP “describes the planned analysis of study objectives … it describes what variables and outcomes will be collected and which statistical methods will be used to analyze them [1]”. National regulatory agencies around the world require SAPs to be submitted when considering drugs, biologics, and devices for approval. The SAP is meant to supplement the protocol and provide richer detail for all prospectively planned statistical analyses. In addition, it defines the population(s) and time point(s) used for each analysis. It defines details such as multiplicity control, sensitivity analyses, methods used to handle missing data, subsets analyses prospectively identified, and the specific analyses performed on each subset of interest. For those populations defined, the SAP should provide clear rules for who is included in each analysis population.

The SAP functions as contract between the study team and the potential consumers of their research [2]. It identifies analyses described in the protocol and ensures there is sufficient detail so prospectively defined methods can be precisely executed. While there may always be post-hoc/exploratory analysis after data is collected, the SAP is used to identify all primary, secondary, and pre-specified exploratory analysis and the precise methods to be used for those. The situation where further detail is required after unblinding of the data is one to be avoided as it may allow for the introduction of bias if the investigators, who have now seen the unblinded trial data, must be approached to provide clarity.

One key goal of a well-written SAP is reproducibility. The standard for this reproducibility can be measured by this idealistic exercise: if multiple statisticians have access to (1) the analysis dataset and (2) the SAP, they would all conduct very similar to the same analyses, and ideally produce the same results. While the trial protocol may describe all primary and secondary endpoints and analyses, the SAP is the place where greater technical detail can be provided to the target audience of statisticians and statistical programmers, in order to achieve this high level of reproducibility.

As the vehicle for key findings and solid science, the SAP is a foundational document to reproducible research. Given the data and the SAP, there should be a very limited number of subjective decisions necessary at the time of the analysis. This ought to help the resulting analyses have the highest possible integrity. Any analysis not prospectively defined in the SAP should be clearly noted as post hoc. Likewise, any analysis that differed in any way from the prospectively planned analysis described within the SAP should be noted with the difference and its rationale.

### 1.2 Create the SAP when creating the trial protocol

The SAP cannot be completed before the protocol is completed. The SAP must be completed before the study is unblinded (in a blinded trial) or the PI or statistical team has access to the accumulating data (in an unblinded trial). For years, conventional wisdom maintained an SAP ought to be finished “after the protocol is finalized and before the blind is broken” [2]. In recent years, many have adopted a best practice that an SAP ought to be finalized before the first patient is enrolled. In some cases, however, protocols that are finished, funded and frozen without an SAP are not clear and detailed enough to carry out a true prospective analysis. One approach to avoid this is to prepare and complete the SAP in parallel with the protocol. Rather than delay, “a statistical analysis plan should be prepared at the time of protocol development [3]”.

There are significant benefits to completing the SAP while the protocol is being completed. The protocol clarity needed by the statistician for the SAP can act as a catalyst to unearth design flaws in a protocol that is ‘in development’. This identification of design flaws is secondary to the primary goal of completing an SAP. This secondary effect is a significant benefit to creating the SAP at the time of the protocol. Evans and Ting confirm that secondary uses of an SAP are valid, including describing the SAP as: “a pseudo-contract between the statisticians and other members of the project team” and “… a communication mechanism with regulatory agencies [2]”. Those who do not complete the SAP concomitantly with the protocol lose a unique opportunity to find study design flaws. Otherwise, those flaws can live in the design until found by the trial implementation team on site or during the data analysis phase. If uncovered during the data analysis phase, it may suddenly dawn on the PI team that the research question cannot be answered with the trial that was implemented. If a trial is implemented to answer a statistically well-defined research question, then “consideration of the statistical methods should underpin all aspects of an RCT, including development of the specific aims and design of the protocol [3]”.

An additional benefit of co-developing the protocol and SAP is the resulting increase in likelihood the study will end informatively. Deborah Zarin and colleagues created the term “uninformative clinical trials” in 2019 [4]. Uninformativeness is a type of research inefficiency. “An uninformative trial is one that provides results that are not of meaningful use for a patient, clinician, researcher, or policy maker [4]”. They describe one potential driver of uninformativeness as when a “study design is pre-specified in a trial protocol, but [the] trial is not conducted, analyzed or reported in manner consistent with [the] protocol [4]”. There is established evidence of SAPs being at odds with their protocol pair at study publication [5,6]. An obvious method to ensure against this possible disconnect is to finalize the SAP during the development of the study protocol.

### 1.3 Engage biostatisticians with domain experience

There are unique characteristics and statistics that tend to repeat within specific types of trials. Examples of these specific types of trials include those with particular pathologies (e.g., cancer, malnutrition), interventions (e.g., vaccines, digital health applications), or study designs (e.g., enrichment, cluster randomized, or challenge trials). RCTs touching different domains will need to apply statistical techniques specific to those domains. For example, in the case of human challenge trials, the SAP would need to include a model for estimating infection fatality risk. For example, recently one team created a Bayesian meta-analysis model to estimate infection fatality in COVID-19 human challenge trials including young participants [7]. In cluster randomized trials, handling the similarities in outcomes amongst participants inside and outside of clusters—referred to as intracluster correlation—adds particular complexities to estimating proper sample sizes [8]. Employing biostatisticians with experience in creating statistical analysis plans for particular trial varietals increases the likelihood of success of those trials.

### 1.4 An excellent SAP rewards the principal investigator (PI) and team

There are a number of secondary positive effects of having a solidly constructed and thorough SAP. These include, but are not limited to:

1. Regulatory Review. If the PI knows there will be a path to engage regulators for approval, or pivots to that decision later, the quality of the SAP will be paramount. Since one cannot re-write or create the SAP post hoc, the commitment to SAP excellence must happen up front.
2. Ethics Committee/Institutional Review Board. Although the SAP is not a core document that an Ethics Committee or Institutional Review Board might use to make key decisions about a study, it might be useful to the members of such groups. In this case, a high-quality SAP will speed approval, while raising the credibility of the PI team.
3. Unanticipated Review. If there is some heavy scrutiny of the RCT, either for positive or negative reasons, requests may emerge for the SAP. If that SAP is reviewed more widely and the stakes are high, the PI team and SAP authors would want a complete, high-quality document.
4. Future Re-Use. With a high-quality SAP, it might be a useful template for future studies. Because it includes a number of endpoints or populations you may use in future studies, regular updates will keep it fresh. This will save time in writing new SAPs from scratch each time.
5. Funding Asset. Showing a thorough and professional SAP enables funders, donors, and sponsors to give grant funding with confidence. As they compare research teams, it is in places like the SAP where the ground truth about capabilities shines through.
6. Future Readiness. More funders, donors, and sponsors are realizing the value of early SAPs for informativeness. It is likely that SAPs will increasingly be requested or be requested pre-funding. Excellent SAPs and the discipline it takes to maintain their creation will make it easier when those shifts occur. Readiness for publication will be higher, as more and more high impact journals are requiring Protocols and SAPs as online appendices during publication.

To realize these benefits as well as the primary goal of ensuring transparent, objective, rigorous, reproducible research, one or more biostatisticians must be engaged to create an SAP. While some biostatisticians write and edit SAPs frequently and are ‘living’ in the world of trial design, more of them are not. Those who are new to SAPs or are irregularly writing such documents could benefit from current best practices and refreshers of fundamentals. Fortunately, a number of contemporary peer-reviewed publications have included SAP checklists. These checklists are designed to ensure SAP authors include all necessary and best practice items [1,2,9]. In addition to these SAP creation checklists, a recent checklist includes items that a biostatistician might look for when reviewing an SAP on behalf of donors, funders, or sponsors [10].

## 2 Materials and methods

The template that follows is organized as an example SAP, with guidance included. While all clinical trials and prospective studies are different, this document describes sections that should or may be desirable to produce a document that will guide objective analyses at the project's conclusion. Not all sections mentioned here will be necessary for all trials or studies and some may be omitted. Furthermore, there may be unique aspects of a study not contained herein that may be required for studies using novel methodology or having uncommon characteristics. The key is that the SAP must contain detailed descriptions of all study populations, endpoints, and preplanned analyses to maximize study integrity and eliminate or limit the subjectivity required in the study's analysis phase. The SAP template herein includes examples from a number of different trials and trial types. This was necessary to provide a wider breadth of examples, and for examples with richer content.

No human participants were included nor involved in this work. As no humans nor animals were involved, there was no opportunity for informed consent, and no ethical approval was sought.

## 3 Results

While all clinical trials and prospective studies are different, this document describes sections that should or may be desirable to produce a document that will guide objective analyses at the project's conclusion. Too often, an SAP contains large chunks of text copied and pasted from the protocol. In some places, e.g., inclusion/exclusion criteria, this may be appropriate. Otherwise the SAP should be viewed as a place to add detail. Therefore, when copying from the protocol, consider whether technical details can or should be added to guide the statistical team at the time of analysis.

The following document is organized as an example Statistical Analysis Plan. Not all sections mentioned here will be necessary for all trials/studies and some may be omitted. Furthermore, there may be unique aspects of a study not contained herein that may be required for studies using novel methodology or having uncommon characteristics. The key is that the SAP contain detailed descriptions of all study populations, endpoints, and preplanned analyses to maximize study integrity and eliminate or limit the subjectivity required in the study's analysis phase.

*Descriptive text defining the aims or providing descriptive detail for each section is found in italics and is meant to guide the SAP authors and it not meant for inclusion in an SAP.***Bold underlined text are examples meant to be replaced by authors with their protocol-specific text**. Other black text is meant as example description and may be kept entirely, edited as necessary, or replaced in its entirety.

## 4 Statistical analysis plan

**Table:** 

**Study Title:**	**Insert the study title of the protocol exactly as presented in the protocol**
**Study Registration Number**	**Insert the study/trial registration number exactly as presented in the protocol**
**Sponsor**	**Sponsor Name**
	**Sponsor Street Address**
	**City, State or Province, Postal Code, COUNTRY**

**Intervention**	**This may be a drug product/vaccine name as described in the protocol or the brief name of the health policy intervention**
**Protocol Number:**	Protocol Number from Protocol. *This Protocol Number refers to a particular version of the protocol referenced by this SAP or associated with this SAP

**Phase:**	**Phase 1, Phase 2, Phase 3, Phase 4 if applicable - Taken from Protocol**

**Statistical Analysis Plan Version**	Version **1.0** *If the SAP is updated after protocol changes this is incremented*

**Statistical Analysis Plan Date**	**Date of Version or Version Update**

**Author**	**Name**
	**Company**
	**Street Address**
	**City, State or Province, Postal Code, COUNTRY**

## 5 Statistical analysis plan approval signature page

**Table:** 

AUTHOR:	**Name:**
	**Position/Title:**
	**________________________________________________**
	**Signature Date**
	*If multiple authors, add additional signature lines.*
APPROVED BY:	**Name:**
	**Position/Title:**
	**________________________________________________**
	**Signature Date**
	**Name:**
	**Position/Title:**
	**________________________________________________**
	**Signature Date**

## 6 SAP revision history

Each time the SAP is given a new version number/the version is incremented, add here a date of the new version, the name of the primary author of the changes, a summary list of changes made, the reasons for those revisions, and any other information that seems suitable to record. Add to each row or entry the estimated number of weeks prior to the first interim analysis the revision was made.

SAP revisions may be aligned with specific protocol revisions as well. Each version of the SAP should reference the latest version of the protocol to which it is aligned.

## 7 SAP roles

**SAP Primary Author: ____________________ (This is the name of the person directly writing most of the document.)**

**Senior Statistician: _______________________ (This is the name of the person who is the most organizationally senior who would actually read and sign off on and be accountable for the correct approaches being included.)**

**SAP Contributor ([Role]): _________________ (This is someone on the team who contributed to the SAP but did not do most of the writing nor are they the senior statistician.)**

**SAP Contributor ([Role]): _________________ (This is someone on the team who contributed to the SAP but did not do most of the writing nor are they the senior statistician.)**

## List of abbreviations

This list typically follows the terms from the protocol that are used in the SAP plus additional terms that are specific to the SAP that are statistical in nature, i.e., MMRM = Mixed Model Repeated Measures. The list below is offered as an example; if you want to use it, please remove terms not used in the protocol, and replace it with your terms.

**Table:** 

AE	Adverse Event
ATC	Anatomical Class (from WHODRUG dictionary)
CRF	Case Report Form
CI	Confidence Interval
CS	Clinically Significant
CSR	Clinical Study Report
CTCAE	Common Toxicity Criteria for Adverse Events
CV	Critical Variable/Coefficient of Variation
ECG	Electrocardiogram
GCP	Good Clinical Practices
ICH	International Committee for Harmonisation
IQR	Interquartile Range
ITT	Intent to Treat
MMRM	Mixed Model with Repeated Measures
PP	Per Protocol
SAE	Serious Adverse Event
SAP	Statistical Analysis Plan
SAS	Statistical Analysis System
SD	Standard Deviation
SOC	System Organ Class
TEAE	Treatment Emergent Adverse Event
WHODRUG	World Health Organization Drug Reference Listing

## 1 Introduction

Example wording to explain the purpose of this SAP. This should lay out the background, rationale, hypotheses, and objectives of the study and may be similar to the intro to the protocol.

The primary objective of this study is to assess the efficacy and safety of **Product Name or Healthcare intervention strategy** in the treatment of **disease name in target population**.

This document outlines the statistical methods to be implemented during the analyses of data collected within the scope of **Trial Group**'s **Protocol Number** titled “**Protocol Title***”.*

This Statistical Analysis Plan (SAP) was prepared in accordance with the **Protocol, Protocol Number**, dated **add date**. *(Original protocol version & date from which first SAP was created)*.

This SAP was modified to be in accordance with the protocol revision(s) **Protocol Number** dated **Date modified***. (Protocol revisions which necessitated updates to SAP)*.

The purpose of this Statistical Analysis Plan (SAP) is to provide a framework in which answers to the protocol objectives may be achieved in a statistically rigorous fashion, without bias or analytical deficiencies, following methods identified prior to database lock. Specifically, this plan has the following purposes:

• To prospectively outline the specific types of analyses and presentations of data that will form the basis for conclusions.
• To explain in detail how the data will be handled and analyzed, adhering to commonly accepted standards and practices of biostatistical analysis. Any deviations from these guidelines must be substantiated by sound statistical reasoning and documented in writing in the final clinical study report (CSR).

Because the SAP is easier to update than the protocol, there may be situations where the SAP is updated later and deviates from the statistical methods described in the protocol. A summary statement should be included to cover this situation indicating the SAP takes precedent when the protocol and SAP deviate:

The analyses described in this analysis plan are consistent with the analyses described in the study protocol. The order may be changed for clarity. If there are discrepancies between the protocol and SAP, the SAP will serve as the definitive analysis plan.

Any analysis performed not prospectively defined in this document will be labeled as post hoc & exploratory.

If a substantive change occurs after the final protocol version it may be included such as:

During the course of data collection, while randomization assignment was blinded, it became evident the primary outcome was heavily skewed right. Therefore, while the protocol cites a regression model for the primary outcome with unity link, the SAP is updated to include a log transformation followed by the same regression model.

## 2 Overview & Objectives of Study Design

This is used to give a brief synopsis of the study design. Typically, this can be from the synopsis in the protocol. Should include study design, dose, phase, and patient population. Authors should confirm that each objective is aligned with one or more study endpoints.

This example is for an oncology product.

This is a multicenter, double-blind, randomized study with a phase 2 portion and a phase 3 portion. Approximately X patients will be enrolled in this study. The phase 2 portion will be open label with all patients receiving study drug at one of two doses.

In Phase 2, patients only with advanced or metastatic NSCLC after failing standard therapy will be enrolled.

In Phase 3, patients with one of the following conditions will be enrolled:

1) advanced or metastatic breast cancer, who have failed ≥1 but <5 prior lines of chemotherapy; advanced or metastatic NSCLC after failing drug xxx-based therapy; or
2) hormone refractory (androgen independent) metastatic prostate cancer.

The eligibility of all patients will be determined during a 28-day screening period.

Phase 2:

Approximately X patients with advanced and metastatic NSCLC will be enrolled. Patients are randomly assigned, with xx patients enrolled in each arm, with the arm designation and planned intervention as follows:

Arm 1: Arm 1 Description, Dosing strategy
Arm 2: Arm 2 Description, Dosing strategy

The study will be temporarily closed to enrollment when Z patients have been enrolled and completed at least 1 treatment cycle in each arm in phase 2. The Sponsor will notify the study sites when this occurs.

Once the study is temporarily closed to enrollment in phase 2, a PK/PD analysis will be performed to determine the RP3D. The PK/PD analysis will be done by an independent party **(may define 3**rd **party here)** at the time 40 patients in Phase 2 have completed at least Cycle 1. This analysis will be blinded to the study team.

Remember all newly introduced abbreviations used above (e.g., RP3D and PK/PD) need to be in the list of abbreviations.

Phase3:

Phase 3 will not begin until RP3D has been determined based on the phase 2 PK/PD analysis as mentioned above. The dose chosen as the RP3D will constitute one arm and active control the other.

Approximately YYY patients are planned to be enrolled in the Phase 3 with one of the following diagnosis: **Put in conditions for enrollment– this is in the protocol as inclusion/exclusion criteria and should match.**

Patients will be randomly assigned with equal probability (1:1 ratio), with the arm designation and planned intervention as follows:

Arm 1: **Describe Arm 1, e.g., the RP3D identified in Phase 2.**
Arm 2: **Describe Arm 2, e.g., the standard of care/control**

For multi-stage seamless trials, it is necessary to define whether data from the initial stage will be combined with data from the follow stages, and if so how.

Data from all patients receiving the RP3D DRUG A dose in Phase 2 and Phase 3 will not be pooled for assessing the primary and secondary study endpoints. Phase 2 is for dose selection. Phase 3 will serve as independent validation and comparison of the chosen dose to active control. Therefore Phase 3 data will be analyzed separately. The primary results will be calculated only from patients enrolled in Phase 3 that have concurrent controls.

Rescue Treatment or other treatments or procedures:

This is usually detailed in the protocol and those details should be presented here if appropriate.

Section 14 of this SAP has further details regarding the schedule of events.

### 2.1 Phase 2 objectives

Objectives should be stated here and should match the objectives in the protocol.

Primary objective:

• To establish the Recommended Phase 3 Dose (RP3D) based on PK/PD analysis.

**Primary efficacy pharmacodynamic objective**:

• To assess DSN in treatment Cycle 1 in patients treated with Dose 1or with Dose 2. Neutrophils count will be assessed at baseline; Pre-dose during Cycle 1, Day 1, 2, 5, 6, 7, 8, 9, 10, 15.

**Primary Safety Pharmacodynamic objective:**

• To assess blood pressure semi-continuously with 15-min intervals, starting 15 min pre-dose and lasting 6 h after start of infusion with drug xxx or drug yyy.

**Secondary objectives:**

• To characterize the pharmacokinetic profile of Dose 1and Dose 2
• To characterize the exposure-response relationships between measures of drug xxx exposure and pharmacodynamic endpoints of interest (e.g., duration of severe neutropenia [DSN]).
• To characterize the exposure-safety relationships between measures of drug xxx exposure and safety events of interest.

**Exploratory objectives:**

• To assess CD34^+^ at baseline, Days 2, 5, and 8 in Cycle 1 and Day 1 in Cycle 2
• Quality of Life as assessed by EORTC QLQ-C30 and EQ-5D-5L
• Disease Progression

**Safety objectives:**

• Incidence, occurrence, and severity of AEs/SAEs
• Incidence, occurrence, and severity of bone pain
• Systemic tolerance (physical examination and safety laboratory assessments)

### 2.2 Phase 3 objectives

**Primary objective:**

• To assess DSN in treatment Cycle 1 in patients with advanced or metastatic breast cancer, who have failed ≥1 but <5 prior lines of chemotherapy; advanced or metastatic non-small cell lung cancer (NSCLC) after failing DRUG-based therapy. Neutrophils count will be assessed at baseline; Pre-dose during Cycle 1, Day 1, 2, 5, 6, 7, 8, 9, 10, 15.

**Secondary objectives:**

• To assess the effects of DRUG A versus DRUG B in patients with advanced or metastatic breast cancer, who have failed ≥1 but <5 prior lines of chemotherapy; advanced or metastatic NSCLC after failing DRUG-based therapy; or hormone refractory (androgen independent) metastatic prostate cancer:
  − Incidence of Grade 4 neutropenia (ANC <0.5 × 10^9^/L) on Days 8 and 15 in Cycles 1 to 4
  − Incidence of FN (ANC<0.5 × 10^9^/L and body temperature ≥38.3 °C) in Cycles 1 to 4
  − Neutrophil nadir during Cycle 1
  − Incidence of documented infections in Cycles 1 to 4
  − Incidence and duration of hospitalizations due to FN in Cycles 1 to 4
  − Health-related Quality of Life (QoL) questionnaire evaluated with European Organization for Research and Treatment of Cancer (EORTC) QLQ-C30 and EQ-5D-5L
  − Use of pegfilgrastim or filgrastim as treatment with neutropenia
  − Incidence of antibiotic use
  − Incidence of docetaxel dose delay, dose reduction, and/or dose discontinuation

**Safety objectives**.

• Incidence, occurrence, and severity of AEs/SAEs
• Incidence, occurrence, and severity of bone pain
• Systemic tolerance (physical examination and safety laboratory assessments)

## 3 Sample Size Justification

This section should contain the complete details for the justification of the sample size. This should include all details and assumptions necessary so the sample size could be independently replicated based upon provided information.

Many organizations, agencies and funders are increasingly encouraging simulation to be used for power calculations, even for designs that may have closed for sample size calculations. Simulation enables various sensitivity analyses such a sensitivity to violated assumptions, to missing data, etc. If simulation was used to calculate the sample size/power, then a reference should be made to archived simulation code. If manageable, the code could be included in a later portion of the SAP or provided elsewhere, e.g. reference to GitHub.

Note here also the phase 2 is a convenience sample and that is stated along with no hypotheses are being tested.

Phase 2 Sample Size Justification.

In the Phase 2 portion of this study, 30 patients with advanced or metastatic NSCLC will be enrolled. It is not powered for testing any statistical hypotheses but a standard sample size for this type of study to support Pk/Pd analysis. No formal hypotheses are being tested in the Phase 2 portion of this study.

Phase 3 Sample Size Justification.

Approximately 150 patients are planned to be enrolled with 1 of the following diagnoses: advanced or metastatic breast cancer, NSCLC, or HRPC. A sample size of 75 patients in each of the treatment arms DRUG A versus Standard of Care, with matching placebos achieve at least a 90% power to reject the null hypothesis of 0.65 day of inferiority in DSN between the treatment means with standard deviations of 0.75, at a significance level (alpha) of 0.05 two-sided two-sample zero-inflated Poisson model. Simulation code to confirm the power for N = 150 patients is contained in a later section.

Another example that uses simulation.

Phase 3 Sample Size Justification.

Negative binomial regression is used to test whether the intervention decreases the need for medical services in the following six months. Assuming medical service utilization will decrease from an average of 3 to 1.5, with SD = 1.25 x the mean for each group, 67 patients per group, 134 total, offers 90% power at the two-sided a = 0.05 level.

**Figure:**
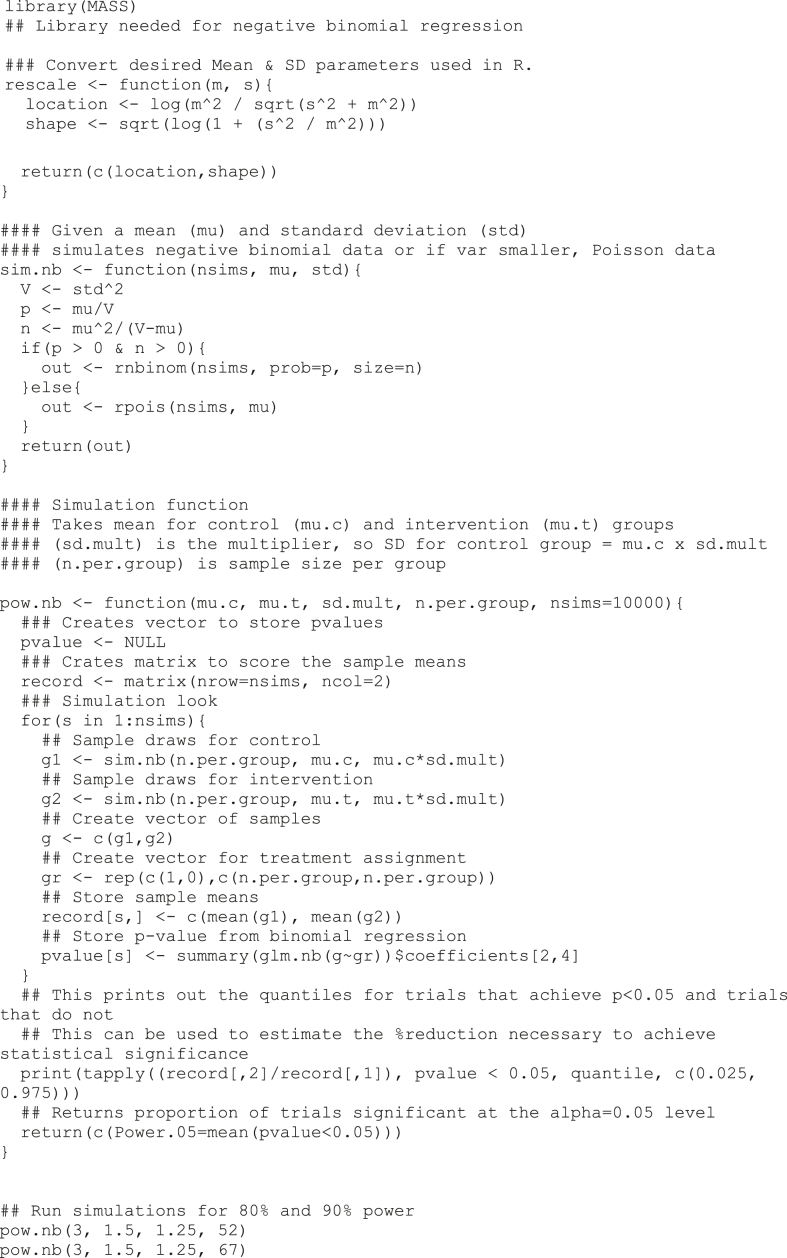

## 4 Randomization, stratification, blinding, and Replacement of Patients

Here, a description of the randomization procedures for the study are needed. If open label, state the same. If randomized, stratified, or any other type of grouping, that needs to be made clear. Also, address if this is a fixed randomization or dynamic as from a central IVRS/IWRS system.

This section should also contain specific information if patients are stratified, and if so how, and details on whether block randomization was performed, along with block size.

Also, if patients withdraw after consent and confirmed eligibility but before randomization, this section may contain information on whether such patients may be replaced.

Patients will be identified by a patient number assigned at the time of informed consent.

### 4.1 Treatment assignment

Patients will be stratified based on his or her diagnosis. Strata 1 and 2 are:

1) advanced or metastatic breast cancer, who have failed ≥1 but <5 prior lines of chemotherapy; advanced or metastatic NSCLC after failing drug xxx-based therapy; or
2) hormone refractory (androgen independent) metastatic prostate cancer.

Patients will be randomized using IVRS/IWRS to 1 of the following treatment groups:

**Phase 2 (10 patients in each arm):**

Arm 1*:* Describe treatment arm.

Arm 2: Describe treatment arm.

No blocking is used in Phase 2.

**Phase 3 (75 patients enrolled in each arm):**

Arm 1: Describe Arm.

Arm 2: Describe Arm.

Random blocks of 4 or 6 are used within each strata in Phase 3.

### 4.2 Rescue treatment/other treatments/dose escalation/other procedures are related

*Describe here any other treatments/procedures that may be administered to the patient that result from efficacy/lack of efficacy or safety issues*.

For example, for a pain trial.

A patient will be considered censored if they require opioids for rescue treatment. The patient will continue to be followed for safety outcomes, but efficacy assessments will terminate and will be counted as missing from that point forward. Such patients will not be replaced in the trial.

## 5 Definitions of Patients Populations to be analyzed

This section should clearly define all potential populations to be used in the data analyses. Each data analysis should then reference the population(s) to be used.

### 5.1 Analysis Sets of Phase 2

#### 5.1.1 Intent-to-treat analysis set (ITT)

The intent-to-treat analysis set for Phase 2 is comprised of all Phase 2 patients that have been randomized.

The analysis of all endpoints, unless noted otherwise, will be conducted on the intent-to-treat analysis set.

#### 5.1.2 Safety analysis set

The safety analysis set will include all patients who receive one or more doses of study drug.

#### 5.1.3 Per Protocol Analysis Set (PP)

Patients who qualify for the ITT population who complete the study period without a major protocol deviation will be included in the PP analysis. Expected major protocol deviations include:

Also include any other circumstances which would preclude a study subject from inclusion in the per protocol population. Examples include:

1. Rescue therapy requiring opioids or other protocol prohibited medications
2. Primary outcome measures out of window
3. Subjects who did not meet all inclusion exclusion criteria

#### 5.1.4 Interim Analysis Set

If the design calls for an interim analysis, define which population will be used. Usually this can denote the ITT or PP analysis sets described above. Sometimes it may include a unique set.

Should also include how it is capped, e.g., ‘first 30 patients enrolled who reach their 3-month endpoint’, or ‘first 30 patients to reach their 3-month endpoint.’

The interim analysis will use the intent-to-treat population but include only patients who have complete primary endpoint data at the time of the interim analysis.

A sensitivity analysis will be performed using the per protocol population, including only subjects with complete primary endpoint data, at the time of the analysis, and presented to the DMC.

#### 5.1.5 Pharmacokinetic Analysis Set

Describe the conditions of the patient population utilized the PK Set.

All subjects who received at least 1 dose of any study and had at least 1 PK sample collected will be included in the PK analysis set. These subjects will be evaluated for PK unless significant protocol deviations affect the data analysis or if key dosing, dosing interruption, or sampling information is missing. For phase 3, PK samples may be collected with a schedule of collection based on the emerging data from Phase 2. Population pharmacokinetic modeling will be utilized to analyze the PK data, and optimal sampling approaches will be used to determine the PK time points for Phase 3.

#### 5.1.6 Pharmacodynamic Analysis Set

Describe the conditions of the patient population utilized in any PD Set.

All patients who had blood pressure and DSN collected at any time during the study will be included in the PD analysis set. For phase 3, PD data may be collected with a schedule of collection to be confirmed based on the emerging data to be determined. Exploratory PK/PD and exposure-response analyses will be conducted to evaluate the effects of DRUG A on safety and efficacy endpoints. Details of these analyses will be summarized in the statistical analysis plan and may be reported outside of the main clinical study report.

### 5.2 Analysis Sets of Phase 3

#### 5.2.1 Intent-to-treat analysis set

The intent-to-treat analysis set for Phase 3 is comprised of all Phase 3 patients that have been randomized.

The analysis of all endpoints, unless noted otherwise, will be conducted on the intent-to-treat analysis set.

#### 5.2.2 Safety analysis set

The safety analysis set will be the same as the intent-to-treat analysis set for Phase 3.

#### 5.2.3 Per Protocol Set

Patients who qualify for the ITT population who complete the study period without a major protocol deviation will be included in the PP analysis. Expected major protocol deviations include:

Also include any other circumstances which would preclude a study subject from inclusion in the per protocol population. Again, there should be sufficient detail in the SAP so that multiple people reviewing the data and the SAP would make the same judgement on who is and is not eligible for the PP set.

#### 5.2.4 Modified intent-to-treat analysis set

Some studies include a modified intent-to-treat analysis which is a minor modification to the ITT analysis.

The intent-to-treat analysis set for Phase 3 is comprised of all Phase 3 patients that have been randomized in the study and have received at least one dose of study medication.

#### 5.2.5 Interim Analysis Set

If the design calls for an interim analysis, define which population will be used. Usually this can denote the ITT or PP analysis sets described above. Sometimes it may include a unique set.

Should also include how it is capped, e.g., ‘first 30 patients enrolled who reach their 3-month endpoint’, or ‘first 30 patients to reach their 3-month endpoint.’

The interim analysis will use the intent-to-treat population but include only patients who have complete primary endpoint data at the time of the interim analysis.

A sensitivity analysis will be performed using the per protocol population, including only subjects with complete primary endpoint data, at the time of the analysis, and presented to the DMC.

## 6 Endpoints

This section describes the primary, key secondary (if any), secondary, and exploratory endpoints and any safety endpoints that are tracked.

This section can be used to provide greater detail, description, or references for endpoints. For example, if the primary endpoint is a composite endpoint (e.g., MACE events), the set of conditions defined the presence of absence of the endpoint should be included. The primary endpoint section below provides an example.

Furthermore, this is the section where you would provide detail on how to calculate derived endpoints, e.g., if a set of survey questions were used to provide a composite score (e.g. HAQ-DI). The key secondary endpoint section provides an example.

Again here, authors should confirm that each objective is aligned with one or more study endpoints.

### 6.1 Primary endpoint

The primary endpoint is the time to first Major Adverse Cardiac Event (MACE). MACE events include the presence of any one or more of the following:

1) Non-fatal stroke
2) Non-fatal myocardial infarction
3) Heart failure leading to hospital admission
4) Ischemic cardiac events
5) Peripheral vascular disease leading to hospital admission
6) Cardiovascular death

Patients experiencing multiple events will have their first, not most severe, MACE event count toward the primary endpoint. All potential MACE events will be reported and sent to the blinded Clinical Events Committee (CEC). The CEC will have access to all clinical information except treatment assignment. If a patient experiences no event their days from randomization to last known office visit will be considered their censored time without an event.

### 6.2 Key Secondary Endpoints

The following is an example of a derived endpoint. A 20-question survey is used to produce a single score for Rheumatoid Arthritis. The endpoint is defined, described, and the analytical method detailed for its calculation. Survey instruments may have missing data for select questions. Ideally this section details how missing data is internally handled with in the calculation of the summary score or cites the paper that describes how piecewise missing data is handled.

The functional status of the subject will be assessed by means of the Disability Index of the Health Assessment Questionnaire (HAQ-DI). This 20-question instrument assesses the degree of difficulty a person has in accomplishing tasks in 8 functional areas: (1) Dressing and grooming, (2) Arising, (3) Eating, (4) Walking, (5) Hygiene (6) Reach, (7) Grip, and (8) Common Daily Activities.

Each functional area contains at least 2 questions. For each question, there is a 4-level response set that is scored from 0 (without any difficulty) to 3 (unable to do). If aids or devices or physical assistance are used for a specific functional area the maximum response of this functional area is 0 or 1 the according value is increased to a score of 2.

If “other” is marked as an aid or equipment, then this can be assigned to a group of four functional areas and will be handled as an aid or equipment for each of the four functional areas. Therefore, if the maximum score of a functional area is 0 or 1 that value is increased to a score of 2 for each of the four functional areas.

Regarding these corrections, the highest response within each functional area determines the score of that specific functional area. If no questions within a given functional area were answered, no score will be provided for that category (even if answers on aids or equipment are available).

HAQ-DI score is only calculated if there are at least 6 functional area scores available. The average of these non-missing functional area scores defines the continuous HAQ-DI score ranging from 0 to 3. If there are less than 6 functional area scores available, no imputation will be done, and the HAQ-DI will be set to missing for the corresponding assessment.

### 6.3 Secondary endpoints

Include additional secondary endpoints.

The following six secondary endpoints will be reported and compared. Because these are secondary endpoints, no formal multiplicity adjustments are made.

1. Time to first stroke within 1-year
2. Time to first myocardial infarction within 1-year
3. Time to first Heart failure leading to hospital admission within 1-year
4. Time to first Ischemic cardiac events within 1-year
5. Time to first Peripheral vascular disease leading to hospital admission within 1-year
6. Time to Cardiovascular death within 1-year

Each secondary endpoint listed here is tracked independently, e.g., if a patient has a peripheral artery event at 4 months and a fatal stroke at 8 months, the first event would contribute to (5) and the second event would contribute to (1) and (6). The patient would be considered censored for all other analyses after month 8 due to his death.

### 6.4 Exploratory endpoints

Include additional exploratory endpoints with descriptions and derivation instructions if necessary.

### 6.5 Safety endpoints

Detail key safety endpoints. Potentially all AEs will be tracked and reported, and all need not be listed here. But key safety concerns that will be specifically monitored should listed.

## 7 Statistical analyses

### 7.1 General Principles

This is a general section to present the planned descriptive statistics, and what software is going to be utilized to analyze the data, and, if appropriate, what quality control (QC) checks will be utilized to ensure QC on tabulations and analyses. The section should also include the validation process, if present.

Statistical analyses will be reported using summary tables, figures, and data listings. Continuous variables will be summarized with counts, means, standard deviations, medians, confidence intervals, minimums, and maximums. Categorical variables will be summarized by counts and by percentage of patients.

Formal inferential statistical analyses techniques will be discussed in subsequent sections of this SAP.

Individual patient data obtained from the case report forms (CRFs), electrocardiogram (ECG), core laboratory, PK data and any derived data will be presented in by-patient listings sorted by study phase (2 or 3), study center, and patient number.

All analyses and tabulations will be performed using SAS Version 9.3 or higher on a PC platform. Table, listings, and figures will be presented in RTF format. Upon completion, all SAS programs will be validated by an independent programmer. In addition, all program output will undergo a senior level statistical review. The validation process will be used to confirm that statistically valid methods have been implemented and that all data manipulations and calculations are accurate. Checks will be made to ensure accuracy, consistency with this plan, consistency within tables, and consistency between tables and corresponding data listings. Upon completion of validation and quality review procedures, all documentation will be collected and filed by the project statistician or designee.

Missing or invalid data will be generally treated as missing, not imputed, unless otherwise stated.

### 7.2 Major protocol violations

*This section is generally from the protocol but is presented here and if any updates and/or special analyses are to be done, they are described here. Such as analyzing patients who had major deviations where data verification was not possible*. *This may also provide additional detail describing how individual cases are adjudicated to decide if they meet the level of a protocol deviation.*

Major protocol violations will be identified by the clinical study team and provided to Biostatistics prior to database lock. A protocol deviation is any noncompliance with the clinical trial protocol or Good Clinical Practice (GCP). The noncompliance may be either on the part of the patient, the investigator, or the study site staff. All patients with major protocol violations will be listed by study center and patient numbers. A protocol deviation committee consisting of the lead study coordinator, medical monitor and lead statistician will review all cases prior to unblinded. Deviations will be determined without knowledge of randomization assignment prior to database lock.

### 7.3 Patient Enrollment and Disposition

Describe how enrollment data is to be analyzed and summarized.

Patient enrollment by site will be tabulated by treatment arm and overall.

Patient disposition will be summarized by treatment arm and overall. This will include number of patients screened, number of patients who were screen failures with reason, number of patients who consented, and number of patients who were randomized.

The summary will include the number and percentage of patients in each of the defined analysis populations in Section 5 above. In addition, frequency counts and percentages of patients’ reported reasons for ending the study will be summarized.

A listing will be presented to describe patient study arm, date of first and last dose, date of last visit or contact, total number of completed cycles, and the reason for ending the study for each patient.

Listings of inclusion/exclusion criteria responses will also be provided.

### 7.4 Description of Demographic and Baseline Characteristics

Describe the summarization or analysis of this data. Note the study populations used for each analysis are detailed.

A summary of age, gender, race, ethnicity, vital signs, ECOG status, tumor staging, tumor type, and prior: medical surgery, radiotherapy, disease surgery, and chemotherapy (Yes/No); along with the number of prior chemotherapy regimens will be presented.

The categorical (discrete) variables will be summarized using counts and percentages. The continuous variables will be summarized using mean, median, standard deviation, and range (maximum, minimum).

All demographic and baseline characteristics will be listed by study center, and subject number.

These summaries will include patients in the ITT population and PP population. Summary statistics described here will be presented for each study arm and overall.

### 7.5 Medical history

This section may be applicable primarily for regulatory trials. Patient medical histories and pre-existing conditions are typically recorded at baseline and included by randomization assignment for the Safety Analysis Set or ITT set.

Medical history data will be coded by system organ class and preferred term, using the MedDRA dictionary.

Medical history will be summarized by body system for each study arm in the Safety Population. The table will be sorted in alphabetic order by system organ class, as well as by incidence and preferred term, and the statistics n and % will be presented by study arm where: n is the number of subjects who present at least one occurrence of the medical history and % is the percentage of subjects. The denominator used for calculating the percentages will be the total number of subjects included in the Safety Analysis set for each study arm.

### 7.6 Specific Relevant Medical History

This section may be more applicable to registration trials and would be used to summarize analysis of medical history or medical condition that may be germane and specific to protocol and/or analysis of efficacy or safety data. For example, demographics are relevant across all trials, but specific characteristics, attributions of disease, or disease duration are specific to the topic being studies.

Histology of NSCLC, disease status, prior treatment, and best response will be summarized in the Phase 2 Study Population using frequency counts and percentage.

For the Phase 3 study, History of advanced or metastatic breast cancer, who have failed ≥1 but <5 prior lines of chemotherapy; advanced or metastatic NSCLC after failing platinum-based therapy; or hormone refractory (androgen independent) metastatic prostate cancer will be summarized using counts and percentages.

### 7.7 Concomitant medications

Concomitant medications relevant to the study that a patient is taking at trial entry or initiated during trial participation may be summarized here.

All medication data will be coded by drug class and indication, using the WHODrug dictionary. All medication taken prior to the first dose of study drug will be classified as prior medication. All medication taken on or after the first dose of study drug will be classified as concomitant medication. Medications with start and stop date that bracket the date of first dose will be summarized as both prior and concomitant medication.

For the purpose of inclusion concomitant medication tables, incomplete medication start and stop date will be imputed as detailed in Section11. Based on imputed start and stop dates, medications that started on or after date of first dose will be included in the concomitant medications table.

Concomitant medications will be summarized in the Safety Population by giving the number and percentage of subjects by preferred term within each therapeutic class, with therapeutic class and medications in each class sorted in alphabetical order. The total number of drugs in each selected therapeutic class will also be presented, where, for example two drugs each belonging to the same class will only contribute once to the presented count.

All prior and concomitant medications, as well as medical procedures will be listed by study center, and subject number.

For the Phase 3 study, the number and percentage of patients who use antibiotics will be summarized and tested for differences between the two arms using Fisher's exact test. Also, the number and percentage of patients who use DRUG D as treatment for neutropenia will be summarized and tested for differences between the two arms using Fisher's exact test.

### 7.8 Physical examination

Summary methodologies and analyses if appropriate in detail.

### 7.9 Study Drug Exposure

This section explains in detail how the Drug Exposure data will be summarized and, if appropriate, analyzed (i.e., missing doses between dose groups, or dose adjustments within subgroups).

For each study phase, study treatment exposure will be summarized in the Safety Population.

For each treatment arm for each product, the following will be summarized using descriptive statistics by study arm and overall:

• Duration of exposure, calculated as (date of last dose – date of first dose+1).
• Number of cycles received per patient.
• Number of cycles with dose modification and (or) dose delay.
• Reasons for dose deviations from planned therapy.

All study drug administration data will be listed by study center and patient number.

For the Phase 3 study, the number and percentage of patients who have dose delayed, dose delayed, and/or dose discontinued will be summarized by treatment arm and tested for differences between the two arms using Fisher's exact test.

### 7.10 Efficacy analysis

Perhaps the most important section of the SAP. This section contains all necessary details for the primary and secondary analyses. The section should contain the explicit hypothesis tests for all primary and secondary analyses, the completely specified model(s) to be used, the alpha-level or Type 1 error strategies to be incorporated through the hierarchy of statistical tests.

In some cases, it may be beneficial to include example statistical code for the primary and secondary analyses, especially if methodologies are not standard.

The primary efficacy endpoint is the Phase 3 analysis of the Duration of Severe Neutropenia (DSN) in Cycle 1 of treatment. However, to define efficacy analyses in chronological order, Phase 2 analyses are described first.

#### 7.10.1 Phase 2

As shown, the null and alternative statistical hypothesis are described. Furthermore, this section illustrates there may be cases where a separate statistical analysis plan, e.g., for PK/PD analyses, are referenced. This is sometimes the case for specialized analyses which may have been authored by a subcontractor or consultant.

All of the pharmacokinetic and pharmacodynamics efficacy and safety endpoints will be analyzed according to the separate PK SAP.

##### 7.10.1.1 Primary efficacy exploratory analysis

The ANC will be summarized by treatment arm and day. The nadir, the day of the nadir, and the percentage of patients in each treatment arm who have Severe Neutropenia will also be summarized.

In addition, an exploratory analysis to assess DSN in treatment Cycle 1 in patients treated with DRUG A with DRUG B at two doses and DRUG A with DRUG C at two does each at, using the Jonckheere-Terpstra Test for Ordered Alternatives. With this statistical procedure, the null hypothesis of equality among treatment group means will be tested (μ_*j*_'s, *j* = 1, 2, 3, 4)

H_0_: μ_*1*_ = μ_*2*_ = μ_*3*_ = μ_*4*_

against the alternative in which order is specified

H_1_: μ_*1*_ ≥ μ_*2*_ ≥ μ_*3*_ ≥ μ_*4*_

where at least one of the inequalities is strict. The mean indices have the following interpretation: 1 = DRUG A DRUG B dose1; 2 = DRUG A DRUG B dose2, 3 = DRUG A DRUG C dose1 and 4 = DRUG A DRUG C dose2. The statistically significant rejection of the null hypothesis will be interpreted, that there is an ordered alternative of responses as indicated by the alternative hypothesis H_1_.

If rejection of a primary hypothesis leads to secondary hypotheses being tested, this condition should be clearly detailed and the subsequent tests be described. For example:

If the null hypothesis is rejected, then pair wise Wilcoxon tests will be performed to aid in the assessment of which treatment(s) contributed to the rejection of the null hypothesis.

Since the Jonckheere-Terpstra Test for Ordered Alternatives is a non-parametric test, this will be performed in SAS using PROC FREQ with JT option. The DSN for this analysis is calculated as in a further Section and since this is an exploratory, non-parametric analysis, no adjustment will be made for the potential number of 0 values of the DSN. The non-parametric nature of the test factors these into consideration.

The primary efficacy analysis will be conducted for the ITT and PP populations.

##### 7.10.1.2 Exploratory analyses

A second exploratory analysis is to assess CD34^+^ at baseline, Days 2, 5, and 8 in Cycle 1 and Day 1 in Cycle 2. This data will be summarized by Cycle and Day and the differences between the treatment arms will be assessed by day using an Analysis of Variance (ANOVA) with a term for ARM. Pairwise differences will be assessed using pre-defined contrasts.

The primary efficacy analysis will be conducted for the ITT population.

#### 7.10.2 Phase 3

##### 7.10.2.1 Primary analysis

While the protocol is likely to contain near full details for the primary analysis, the SAP offers additional space and should include all details so that the primary analysis is completely replicable and requires no interpretation or additional assumptions. For example, with a simple chi-square test, this will include the precise test used, whether it will include a continuity correction, when (or if) a Fisher's exact test might be used in lieu of the chi-square test, and the conditions that would necessitate the change. It should specifically include the population on which the primary analysis will be conducted.

This section should contain the precise hypothesis to be tested with null (H_0_) and alternative (H_1_) hypotheses, including whether it is a superiority, equivalence, or non-inferiority testing framework. Because it uses a slightly non-standard model, the SAP also provides illustrative code that defines some of the finer statistical aspects of the predefined model. It should also be clear whether tests are one or two-tailed and the alpha-level used for all statistical tests.

The primary hypothesis of the Phase 3 study is to establish the non-inferiority of DRUG A to DRUG B with respect to DSN in Cycle 1. This non-inferiority trial design will utilize a difference (arm 2 minus arm 1) of 0.65 days (non-inferiority margin) in DSN in Cycle 1 as the largest acceptable difference between DRUG A and DRUG B. The non-inferiority test will evaluate the null hypothesis that the difference in means is greater than or equal to 0.65

H_0_: μ_A_ - μ_B_ ≥ 0.65

against the alternative hypothesis that the difference in means is less than 0.65

H_1_: μ_A_ - μ_B_ < 0.65

DRUG A will be considered non-inferior to DRUG B if in Cycle 1, the upper limit of the 2-sided 95% confidence interval for the true difference in mean duration of Grade 4 neutropenia was <0.65 days.

The analysis of this data will assume a Zero Inflated Poisson Model and will conducted using PROC GENMOD as:

**Figure:**
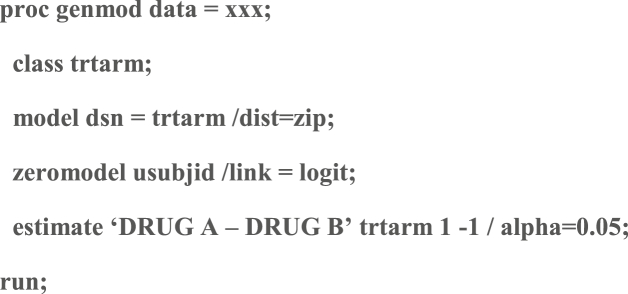

Confidence intervals for the difference will be calculated from the estimate statement and those confidence intervals will be utilized in the assessment of non-inferiority. Also, if the null hypothesis is rejected and non-inferiority is established, then superiority will be tested using the p-value from the above analysis.

The primary efficacy analysis will be conducted for the ITT and PP populations.

Here again when Ho is rejected leading to the conclusion of non-inferiority, superiority is subsequently tested. All potential pre-defied analyses resulting from the result of pre-defined analysis should be clearly stated along with the condition that would lead to their testing.

*Another circumstance which may result in the model not being entirely prespecified* a priori *is when it involves a complicated covariance structure. In such a case the method used to select the covariance structure can be pre-specified. For example, below the primary model is prespecified but the correlation structure of the MMRM model is chosen according to the option that provides the optimal AIC:*

The primary endpoint, change from baseline, will be analyzed using Mixed-effect Model Repeated Measure (MMRM) statistics. The repeated-measures analysis will be based on the restricted maximum likelihood method assuming an unstructured covariance structure to model the within-subject errors. The model will include treatment group (Placebo = 0, Treatment = 1), location (US = 0, OUS = 1), visit (Week 0, 2, 4, 6, 8, 10, and 12), and treatment-by-visit interaction as fixed effects and baseline as a covariate. Treatment effects will be calculated via LSMEANS for each timepoint but the treatment effect at Week 12 will be considered the Primary Efficacy Endpoint. Patient will be considered a random effect.

The data collected after receiving rescue therapy will be set to missing. Therefore, the MMRM analysis assumes a missing-at-random (MAR) mechanism for missing data due to dropout and post-rescue data.

In addition to using an (1) unstructured covariance matrix, the same model will be fit using a (2) compound symmetric covariance matrix and (3) and AR(1) covariance matrix to model the covariance in repeated measures within patient.

Akaike Information Criteria (AIC) will be calculated for each of the three models. The AIC form used will be AIC = 2 K–2 log(L) where K is the number of parameters and L is the log-likelihood. The model with the lowest AIC will be chosen as the primary model and the estimated treatment effect will be reported based upon this model.

The primary efficacy analysis will be reported for the ITT and PP populations.

##### 7.10.2.2 Secondary analyses

The ANC will be summarized for each day within Cycle 1. The neutrophil nadir will be summarized for both treatment arms and tested for difference using a two-sample *t*-test at the 2-sided 0.05 level.

For all four Cycles and all days, the number and percentage of patients who have:

1) Grade 4 Neutropenia
2) Incidence of FN

Will be summarized and tested for differences between the treatment arms using Fisher's exact test.

*Occasionally the chosen statistical test may depend on the final form of the data. This should be detailed in the SAP. For example, if the data is predicted to be normally distributed, but there is concern,* a priori*, regarding this assumption, it can be prospectively defined that a non-parametric test could be used. In such a case the conditions that would lead to the change should be clearly defined and the primary analysis method for if the conditions are or are not met. For example:*

Normality of the primary endpoint will be tested using the Kolmogorov–Smirnov test. If the null hypothesis of normality cannot be rejected, p > 0.05, then a two-sample *t*-test will be used to test the null hypothesis of a difference in means. If p ≤ 0.05 for the K–S test, then normality will be rejected and a Wilcoxon rank sum test will be used to test for a difference in central tendency of the two groups.

##### 7.10.2.3 Exploratory analyses

The statistical methods should be detailed for each exploratory analysis separately. This is also the place to prospectively describe statistical graphics (e.g., histograms, boxplots, barplots, etc.) that will be created. Just like with primary analysis, it is beneficial to define the parameters for the statistical graphics, e.g., bin size for histograms, what categories CDFs may be split by for subsets, etc.

This may oftentimes be a very long section because lots of exploratory analyses may be planned.

During Cycle 1, the nadir for Absolute Neutrophil Count (ANC) will be calculated:

1) If that nadir is not grade 4 in nature (ANC <0.5 × 10^9^/L), then that patient will have a DSN equal to 0 days. That is, the duration is 0 days since the patient did not have severe neutropenia.
2) If the nadir is grade 4 then a regression of ANC vs days will conducted with the first day utilized in the regression being the day the nadir occurred. From this regression, the time (in days) at which the predicted ANC from the regression is ≥ 0.5 × 10^9^/L will be the DSN for that patient.

The regression analysis for each patient will be performed using PROC MIXED in SAS with both a random slope and intercept and, when possible, an unstructured covariance model. If the parameters are not estimable (lack of model convergence) using an unstructured covariance model, then ordinary least squares estimates of the slope and intercept will be performed. The difference will be tested using a Wilcoxon test and confidence intervals will be established using a boot strap methodology.

The results of the Quality of Life assessments will be summarized via descriptive statistics for each treatment group and differences tested using a *t*-test. Since this is exploratory no adjustment for multiple tests will be utilized.

All exploratory analysis will be conducted for the ITT population.

##### 7.10.2.4 Subset analyses

This section should contain all prespecified subset analyses. For example:

The primary, key secondary and secondary analysis will be repeated by.

1. Country
2. Pre-existing conditions
  a. Diabetes Yes vs. No
  b. Prior MI Yes vs. No
3. Age <40 vs. Age ≥ 40
4. Etc.
  If ever a subset has <5% of the total data, analysis of that subset will be omitted.

All analyses will be performed on the ITT populations and reported with p-values and 95% confidence intervals. Results, however, will be viewed as exploratory/hypothesis generating.

In addition to tables for each analysis, a forest plot will be produced for each outcome (primary, key secondary and all secondaries) illustrating treatment effects and 95% CIs by each subset.

##### 7.10.2.5 Sensitivity analyses & missing data

This section should contain all specified sensitivity analyses including sensitivity analyses to evaluate the impact of missing data. For example, to be used with the MMRM methods from above:

A number of sensitivity analyses will be performed to study the effect of missing data on the primary efficacy analysis.

Below are the descriptions for the imputation methods that will be used throughout the efficacy analyses. For example:

The primary efficacy analysis will be performed with observed cases (OC). Missing values remain missing. The primary efficacy analysis will be repeated with the following methods:

(1) For the primary effectiveness analysis at Week 12, multiple imputation will be used to assign a value to those cases with missing data. The full conditional specification method with predictive means matching as described in Berglund & Heeringa (2014) will be used. This method uses all of an individual's known primary outcome measures at Baseline, 2, 4, 6, 8, 10, and/or 12 weeks to impute any missing values.
(2) Last observation carried forward (LOCF). Baseline measurements will not be carried forward to post-baseline. Only post-baseline measurements will be LOCF. For the composite endpoints, the last non-missing post-baseline observation will be carried forward to subsequent visits for each individual component first, and then the composite endpoints using individual components imputed by LOCF will be calculated as described above. If a subject does not have a non-missing observed record for a post-baseline visit, the last post-baseline record prior to the missed visit will be used for this post-baseline visit. If the last non-missing observation prior to the missing visits cannot be determined due to multiple measurements occurring at the same time or the time not available within the same day, the worst outcome will be used for LOCF. If missing components still exist after LOCF, the composite endpoints will be calculated using the same rules as described in OC.
(3) Tipping point analysis. If the primary analysis is statistically significant, a Tipping Point analysis will be used. For patients randomized to treatment who fail to complete the 12-week study period, 0.1 will be subtracted to each of their 12-week primary outcome scores (made worse), while for patients randomized to control who fail to complete the 12-week study period, 0.1 will be added to each of their average last 14 available days (made better), and the analysis repeated. This will be repeated, in increments of 0.1, until the primary analysis fails to be statistically significant at the α = 0.025 level. The increment value at which statistical significance ceases will be reported as the Tipping point.

##### 7.10.2.6 Multiplicity control

Type 1 error control is an important part of a clinical trial. Tight Type 1 error control requires the order of testing and methods for error control to be pre-defined. This increases the integrity and validity of inferences found to be statistically significant.

If multiple primary hypotheses are to be tested, or formal claims are desired for secondary endpoints, then multiplicity (Type 1 error) control should be strictly controlled.

Methods to test multiple hypotheses at once such a Bonferroni method, Hochberg method or Holm's method, should be detailed. Or if a hierarchical testing procedure or hierarchical family of tests is used, it should be clearly defined along with the order of testing.

The primary efficacy analysis will be tested at the one-sided 0.025 level. If it is statistically significance, then the four secondary endpoints will be tested using Hochberg's method.

Hochberg's method is used to control the familywise Type 1 error rate (FWER) among these 4 secondary endpoints. If and only if the primary efficacy objective is met, the Hochberg's step-up procedure (Hochberg 1988) will be used to control the FWER at a 1-sided significance level of 0.025 for the following 4 secondary endpoints:

**Endpoint 1** at Week 12 from Day 0

Test: H_0_: pt - pc = 0 H_A_: pt - pc > 0, using stratified CMH test, as detailed in the secondary analysis section.

**Endpoint 2** Week 12 from baseline where baseline is the day of informed consent

Test: H_0_: pt - pc = 0 H_A_: pt - pc > 0, using stratified CMH test.

**Endpoint 3** at Week 12 from baseline on the day of informed consent

Test: H_0_: pt - pc = 0 H_A_: pt - pc > 0, using stratified CMH test.

**Endpoint 4** at Week 12 from baseline on the day of informed consent

Test: H_0_: pt - pc = 0 H_A_: pt - pc > 0, using stratified CMH test.

The procedure ranks the p-values from the above 4 tests from the least significant (largest p-value, p [4]) to the most significant (smallest p-value, p [1]) and examines the other p-values in a sequential manner until it reaches the most significant one, i.e., p [4] > p [3] > p [2] > p [1].

The decision rule for the Hochberg procedure is defined as follows:

Step 1. If [4] > 0.025, retain H [4] and go to the next step, here H [4] is the hypothesis corresponding to p [4], i.e., the hypothesis with the largest p-value. Otherwise reject all hypotheses and stop.
Step 2. If p [3] > 0.025/2, retain H [3] and go to the next step. Otherwise reject all hypotheses and stop.
Steps 3. If p [2] > 0.025/3, retain H [2] and go to the next step. Otherwise reject all remaining hypotheses and stop.
Steps 4. If p [1] > 0.025/4, retain H [1] otherwise reject it.

The adjusted p-values are calculated as detailed below:

Adjusted p[i] = p(4) for i = 4 other adjusted p[i] = minimum of [adjusted p[i+1], (5-i)*p[i]] for i = 3, 2, 1.

If any adjusted p-value exceeds 1, it is set to 1. Using this procedure, any adjusted one-sided p-value that is < 0.025 is statistically significant and supports a claim for the corresponding endpoint, while any adjusted p-value ≥0.025 is not statistically significant. Both adjusted and unadjusted p-values will be reported.

### 7.11 Safety analysis

Frequently no specific hypotheses are tested for safety. Rather adverse events and their severity (e.g., mild, moderate, severe) are recorded and presented in tabular form. These may be grouped by body system or type (e.g., neurological, GI, etc.).

The Safety analysis set will be used for all safety analysis. Patients will be evaluable for safety analysis if they receive at least one dose of study drug. All subjects receiving a dose of study drug will be included in all safety summaries. The safety data will be presented by study arm in individual listings and summary tables, including frequency tables for adverse events and frequency and shift tables for laboratory variables. All adverse events and abnormal laboratory variables will be assessed according to the NCI CTCAE (v 4.0) grading system. Descriptive statistics will be used to summarize ECOG performance status. Vital signs will be reported in listings. AEs and SAEs will be reported in combined tables. However, SAEs will be tabulated in their own table as well.

All safety information will be listed by study center and subject number.

#### 7.11.1 Adverse events (AEs)

For the final analyses of the safety and tolerance of study drug, all treatment - emergent and overall incidences of adverse events will be summarized by system organ class and by preferred terms (MedDRA). AEs will be considered as treatment - emergent adverse events (TEAE) if onset is on or after the initiation of study treatment. Adverse events with missing onset dates will be summarized as TEAE regardless of severity and relationship to study medication.

The incidence of adverse events by severity and/or CTCAE adverse events grade (mild, moderate, severe, life threatening or death) and by relationship to study drug will be tabulated similarly. Each adverse event will be reported by greatest known severity and by strongest relationship to the study drug.

Each patient will be counted only once within a system organ class or a preferred term by using the AEs with the highest severity grade.

All information pertaining to AEs noted during the study will be listed per patient, detailing verbatim, preferred term, system organ class, start date, stop date, severity, and relationship to study treatment. AE onset will be shown relative (in number of days) to the day of the first study treatment.

For the Phase 3 study, the percentage of patients with an adverse event and the distribution of adverse events across CTCAE grades will be tested for differences between the two treatment arms using Fisher's exact test.

For the Phase 3 study, the number and percentage of patients who have infections will be summarized for each treatment group and tested for differences using Fisher's exact test. Also, the number and percentage of patients who have hospitalizations due to FN will be summarized by cycle and overall for each treatment group and tested for differences using Fisher's exact test.

#### 7.11.2 Serious adverse events

All serious adverse events will be listed by study arm.

#### 7.11.3 Adverse events leading to discontinuation from study

All adverse events leading to discontinuation from study will be listed by study arm.

#### 7.11.4 Deaths

All deaths within 30 days of last study treatment will be listed by study arm. Treatment emergent deaths are those deaths within 30 days of last dose of any study therapy. Early deaths are those deaths within 60 days of the first dose of study therapy.

Treatment emergent and/or early deaths will be tabulated and summarized by treatment groups.

For Phase 3, The difference in the percentage of patients in the percentage of patients of who had treatment emergent deaths and who had early deaths will be tested using Fisher's exact test.

#### 7.11.5 Clinical Laboratory Tests

Safety laboratory data will include clinical chemistries, hematology, and urinalysis. Safety summaries in the form of shift tables for key laboratory parameters showing the number and percentage of patients who experience changes in laboratory parameters during the course of the study (e.g., change from normal to high, based on the laboratory reference ranges) will be displayed. Also shift tables for changes in CTCAE grades will be summarized by counts and percents. If appropriate for specific time points, differences in distributions in shifts between the two treatment arms in Phase 3 will be evaluated using Fisher's exact test.

Descriptive summary statistics (mean, standard deviation, median, minimum, maximum, frequencies, and percentages, as appropriate) for laboratory values will be presented at baseline, the follow-up time points, and change from baseline for each study treatment arm.

All laboratory data, values, units, normal reference range, and out-of-range flags collected in the clinical database will be included in by-patient listings for further medical review.

#### 7.11.6 Vital signs

Vital signs (including temperature, respiratory rate, blood pressure, heart rate, and weight) will be presented descriptively at baseline and for each follow-up time point for each study treatment arm. The number (n), mean, standard deviation, median, range will be presented. Changes from baseline to each time point will also be summarized as well as shifts from normal to abnormal results.

All vital sign parameters will be included in by-patient listings for further medical review.

#### 7.11.7 ECGs

For patients in the Phase 3 study, will be analyzed for QTc prolongation by Fridericia's adjustment to QTc. The average of the three replicates will be used and compared to the average of the three replicates done just prior to the start of the infusion. The incidence of QTC prolongation by either calculation of >30 and > 60 ms will be presented. The incidence of QTc prolongation by either calculation of >480 ms post-infusion will be presented.

All ECGs will be summarized descriptively using N, mean, standard deviation, median, and range for each treatment arm at each visit ECGs are collected (see Section 13).

All ECG parameters will be included in by-patient listings for further medical review.

#### 7.11.8 Other Safety Parameters

The health related QOL EORTC QLQ-C30 and the EQ-5D-dL questionnaires will be summarized for each visit performance status was assessed and as changes from the baseline assessment. Differences between treatment arms will be tested using a *t*-test for each time point where the data is collected.

#### 7.11.9 Population Pharmacokinetic Analysis

This is the spot for details of this analysis if any. If the analysis is detailed using specialized Population PK software a separate PK analysis plan may be needed.

Population pharmacokinetic analyses will be conducted to evaluate the effect of intrinsic and extrinsic factors on the PK of DRUG A and its active metabolite(s), if identified, in humans. Intrinsic factors such as gender, age, hepatic or renal impairment, and race and/or ethnicity and extrinsic factors such as concomitant drugs, herbal products will be assessed in relationship to drug exposure according to FDA Guidance for Industry, “Population Pharmacokinetics”.

Further details of PK analysis will be presented in the PK analysis plan.

## 8 Interim analysis

If an interim analysis(es) is going to be performed describe it here. Include number of patients, alpha level adjustments, statistical methodologies, etc. This may include a choice to reference the methodology described above or provide full detail of the analytical methods at the interim analysis. For example:

The study will incorporate one interim analysis after 100 patients, 2/3 of the total sample size, have reached their primary 12-week outcome. An O'Brien-Fleming stopping rule will be used. The independent statistician will report the results of the interim analysis only to the Data Monitoring Committee.

The primary analysis method described above will be used after 100 patients meeting the ITT criteria have been enrolled and reached their 12-week primary efficacy outcome.

The one-sided p-value from the primary analysis will be compared to a critical value of 0.0071. If the p-value ≤0.0071, the DMC may recommend the trial stop early for overwhelming success. Otherwise, provided no safety concerns, the DMC will recommend the trial continue. If the study continues to the maximum sample size, a critical value of 0.0226 will be used to account for the 0.0071 error spend at the interim analysis.

*Alternatively, some adaptive trials have a separate, smaller Interim Statistical Analysis Plan (iSAP) that may be referenced. This may be done for more complex adaptive trials where a small report is preferred to a section of the SAP. Alternatively, sometimes this is done to blind investigators to details of the interim analysis that could lead investigators to reverse engineer an effect size and become partially unblinded. For example, see Hager* et al. *in the references. Additional file #2 (*https://static-content.springer.com/esm/art%3A10.1186%2Fs13063-019-3254-2/MediaObjects/13063_2019_3254_MOESM2_ESM.pdf*)*

is an adaptive design report specifically detailing the calculations that take place only at the interim analyses.

The study will include a futility analysis and potential sample size re-estimation after ½ of enrolled patients have achieved their primary 12-week outcomes. Full details of the futility analysis and sample size re-estimated are included in the Interim Statistical Analysis Plan.

The futility is a non-binding futility analysis and Type 1 error is conserved even if the futility rule is achieved but not invoked. The independent statistical will provide results to the Data Monitoring Committee (DMC) at the interim analysis.

## 19 Statistical Analysis Changes From the Protocol

Describe changes in methodology that deviate from protocol. There should almost always be none, but if some new methodology is appropriate that was not known at protocol time, that may be described here. For example, if the blinded data was analyzed after the protocol was complete but before SAP completion and database lock. At that time, perhaps it became apparent that the protocol-defined method may not be statistically appropriate for the primary analysis. Two examples:

No changes were made from the original protocol.

Or.

During the course of data collection, while randomization assignment was blinded, it became evident the primary outcome was heavily skewed right. Therefore, while the protocol cites a regression model for the primary outcome with unity link, the SAP is updated to include a log transformation followed by the same regression model.

Also include a statement to cover how necessary, and ideally rare, deviations from the SAP will be communicated.

Any necessary deviations from these guidelines will be.

• documented in the final clinical study report (CSR)
• include the reason the predefined methods here were not appropriate
• detail why the updated methods represent sound statistical reasoning.

## 10 Conventions

The example here is a standard convention that is almost universally accepted. The section should, however, be tailored to each protocol's requirements.

The precision of original measurements will be maintained in summaries, when possible. Means, medians and standard deviations will be presented with an increased level of precision; means and medians will be presented to one more decimal place than the raw data, and the standard deviations will be presented to two more decimal places than the raw data.

Summaries of continuous variables that have some values recorded using approximate values (e.g., lab values < 0.001 or >200) will use imputed values. The approximate values will be imputed using the closest exact value for that measurement. For tables where rounding is required, rounding will be done to the nearest round-off unit. For example, if the round-off unit is the ones place (i.e., integers), values ≥ XX.5 will be rounded up to XX+1 while values < XX.5 will be rounded down to XX.

Percentages will be based on available data and denominators will generally exclude missing values. For frequency counts of categorical variables, categories whose counts are zero will be displayed for the sake of completeness. For example, if none of the patients discontinue due to “lost to follow-up,” this reason will be included in the table with a count of 0. Categories with zero counts will not have zero percentages displayed.

For adverse event incidence tables:

• The order of SOCs presented in tables will be according to the internationally agreed order of SOCs according to MedDRA. Within each SOC, the preferred terms will be shown in alphabetic order.
• Patients who have multiple events in the same SOC and/or preferred term will be counted only once at each level of summation (overall, by SOC, and by preferred term) in the tables. For summaries of AEs by severity, only the highest severity of AE will be counted at each level of summation (overall, by SOC, and by preferred term) in the tables. For summaries of related AEs, patients with more than one related AE will be counted only once at each level of summation (overall, by SOC, and by preferred term) in the tables.

## 11 Standard Calculations

The example here is a standard conventions that is almost universally accepted. The section should, however, be tailored to each protocol's requirements.

Variables requiring calculation will be derived using the following formulas:

Study day – For a given date (date), study day is calculated as days since the date of first dose of study drug (firstdose):

• Study day = date – firstdose + 1, where date ≥ firstdose
• Study day = date – firstdose, where date < firstdose

Days – Durations, expressed in days between one date (date1) and another later date (date2), are calculated using the following formula: duration in days = (date2-date1).

Weeks – Durations, expressed in weeks between one date (date1) and another later date (date2), are calculated using the following formula: duration in weeks = (date2-date1)/7.

Months – Durations, expressed in months between one date (date1) and another later date (date2), are calculated using the following formula: duration in months = (date2-date1)/30.4.

Years – Durations, expressed in years between one date (date1) and another later date (date2), are calculated using the following formula: duration in years = (date2-date1)/365.25.

Minutes – Durations, expressed in minutes between one timepoint (time1) and another later timepoint (time2), are calculated using the following formula: duration in minutes = (time2-time1)/60.

Age – The patient's age is calculated as the number of years from the subject's date of birth to the date of randomization into the study:

• Age = ([Randomization Date - Date of Birth]/365.25).

## 12 Imputation of Dates

Add specific cases and examples if germane to study i.e., disease diagnosis dates.

### 12.1 Incomplete cancer diagnosis

If day is missing, day will be set to 15th of the month, or date of first dose, whichever is earlier. If month and day are missing, month and day will be set to July 1st, or date of first dose, whichever is earlier.

### 12.2 Adverse event

If onset date is completely missing, onset date is set to date of first dose unless end date is before date of first dose, in which case the onset date is set to 28 days prior to end date.

If (year is present and month and day are missing) or (year and day are present and month is missing):

• If year = year of first dose, then set month and day to month and day of first dose unless end date is before date of first dose, in which case the onset date is set to 28 days prior to end date.
• If year < year of first dose, then set month and day to December 31st.
• If year > year of first dose, then set month and day to January 1st.

If month and year are present and day is missing:

• If year = year of first dose and if month = month of first dose then set day to day of first dose date unless end date is before date of first dose, in which case the onset date is set to 28 days prior to end date.
• if month < month of first dose then set day to last day of month
• if month > month of first dose then set day to 1st day of month
• if year < year of first dose then set day to last day of month
• if year > year of first dose then set day to 1st day of month

For all other cases, set onset date to date of first dose unless end date is before date of first dose, in which case the onset date is set to 28 days prior to end date.

### 12.3 Concomitant Medications

If start date is completely missing: start date will not be imputed.

If (year is present and month and day are missing) or (year and day are present and month is missing): set month and day to January 1.

If year and month are present and day is missing: set day to 1st day of month.

If end date is completely missing: end date will not be imputed.

If (year is present and month and day are missing) or (year and day are present and month is missing): set month and day to December 31.

If year and month are present and day is missing: set day to last day of the month.

Any partial dates will be displayed in data listings without imputation of missing days and/or months (e.g., MAR2011, 2009). No other imputation of missing data will be performed.

## 13 Statistical packages

Include here all software products/statistical packages used in creation of simulations for calculating sample sizes, used for other statistical needs in this SAP, as well as the expected statistical package(s) expected to be used in the final analyses or interim analyses.

All analyses and tabulations will be performed using SAS Version 9.3 or higher on a PC platform. R Version 3.1 or higher may be used for statistical graphics.

### 13.1 SAP sample references

References should be provided for unique methodology and/or for subject-specific endpoints, e.g., papers that described a validated endpoint. References may also be provided for software and specific software packages. The following references are not necessary to cite but are frequently cited within SAPs and/or protocols.

1. International Conference on Harmonisation of Technical Requirements for Registration of Pharmaceuticals for Human Use, ICH Harmonised Tripartite Guideline, Statistical Principles for Clinical Trials (E9), 5 February 1998.
2. International Conference on Harmonisation of Technical Requirements for Registration of Pharmaceuticals for Human Use, ICH Harmonised Tripartite Guideline, Structure and Content of Clinical Study Reports (E3), 30 November 1995.
3. International Federation of Pharmaceutical Manufacturers and Associations. Medical Dictionary for Regulatory Activities (MedDRA). Version 14.0 Reston, Virginia, USA; 2008.
4. WHO Collaborating Center for International Drug Monitoring. WHO Drug Dictionary. June 2012 B Format edition. Uppsala, Sweden; 2008.
5. SAS Institute Inc. SAS Version 9.1. Cary, NC, USA; 2002–2003.
6. Hager, D.N., Hooper, M.H., Bernard, G.R. et al. The Vitamin C, Thiamine and Steroids in Sepsis (VICTAS) Protocol: a prospective, multi-center, double-blind, adaptive sample size, randomized, placebo-controlled, clinical trial. *Trials* **20,** 197 (2019). https://doi.org/10.1186/s13063-019-3254-2
7. Berry Consultants. Adaptive Design Report for a Trial of the Virginia Cocktail. Supplementary materials to Hager et al. (above). https://static-content.springer.com/esm/art%3A10.1186%2Fs13063-019-3254-2/MediaObjects/13063_2019_3254_MOESM2_ESM.pdf

## 14 Schedule of events

To make following the sequence of events and timing of patient assessments, the schedule of events from the protocol should be reproduced here. It may be necessary to insert other statistical events into the schedule i.e., interim analyses, second randomization schemes, timing of final analysis, or analysis patient responder status.

## 15 List of Tables, listings, and figures

This section may contain a numbered list of Tables, Listing and Figures to be presented. It is recommended that the primary statistician use partial data without randomization assignments, or dummy randomization assignments, and execute the SAP. This will elucidate if there are uncertainties or ambiguities in the SAP that need to be clarified prior to database lock and breaking of the blind.

Some SAPs, though admittedly rare, may also include pseudocode and table shells or example figures [11].

## 16 Discussion

One weakness of this paper is that it leaves as out of scope the presentation of a complete “exemplar” SAP for a single trial. Fortunately, examples of complete single-trial SAPs can increasingly be found as supplemental materials attached to trial records on clinicaltrials.gov or in supplemental online-only materials for published trials [[11], [12], [13], [14], [15]]. For example, a recent search on clinicaltrials.gov for completed interventional trials with the keyword “malaria” that included an SAP returned 51 records. Alternately, SAPs can sometimes be found as public or global ‘goods’ on open repositories, or via basic internet searches. One consequence of the multiple-trial approach for examples used herein is that it could create conceptual disconnects if table shells, listing shells, or other shells were added to the example SAP. Beyond single complete SAPs, it may be helpful to review at depth both examples of poorly constructed or executed analysis plans, and global standards for analysis publication [16].

Another weakness is the lack of detail on the updating and amendment processes that may occur during the trial. Every time the protocol is updated, the investigators and statistician(s) should evaluate whether SAP changes are also necessary. When the SAP is updated, it should indicate the revision number and ideally contain an easy-to-follow revision history. Oftentimes the original and final SAP are published in supplementary materials, for example Kalil et al.'s recent COVID-19 trial presented both online [17,18].

Further, the paper does not list general keys, tips, and best practices for writing SAPs, nor does it enumerate evidence-based or anecdotal ‘common mistakes’, pitfalls, or risks. Fortunately, a number of contemporary publications identify both positive suggestions and risks, and ought to be consulted to augment this or other practical guides [1,3,5,6,9,16,19,20].

This paper is a novel contribution to scientific literature in part because it is the first time a peer-reviewed, full-length statistical analysis plan (SAP) template, with instructions, is published in full. Previously, guides or sections have been listed and described, but not including the full template needed by new practitioners. This is significant as global health human clinical trial research is increasingly desired by both Global North and Global South stakeholders to be originated and planned within the Global South. Aspiring principal investigators in low-resource settings lack full-length tools that are prevalent amongst Global North contract research organizations, industry, and academic researchers who originate current SAPs. This is compounded by the relative lack of advanced biostatistical education in locations like Africa—where such SAP templates might be expected to exist.

## Ethics approval and consent to participate

Not applicable.

## Consent for publication

Not applicable.

## Availability of data and materials

All data is contained within the manuscript.

## Funding

Partial funding of this work was provided by The 10.13039/100000865Bill & Melinda Gates Foundation. That funder had no role in if any, in study design; in generation of content; in the collection, analysis and interpretation of data; in the writing of the report; and in the decision to submit the article for publication.

## Author's contributions

GS: Conceptualization, Formal Analysis, Investigation, Methodology, Project Administration, Software, Visualization, Writing-Original Draft Preparation.

SD: Conceptualization, Funding Acquisition, Visualization, Writing-Original Draft Preparation.

RM: Validation, Writing-Review & Editing.

JC: Conceptualization, Formal Analysis, Funding Acquisition, Supervision, Validation, Visualization, Writing-Review & Editing.

## Declaration of competing interest

The authors declare that they have no known competing financial interests or personal relationships that could have appeared to influence the work reported in this paper.

## Data Availability

No data was used for the research described in the article.
